# Rho family GTPases: key players in neuronal development, neuronal survival, and neurodegeneration

**DOI:** 10.3389/fncel.2014.00314

**Published:** 2014-10-07

**Authors:** Trisha R. Stankiewicz, Daniel A. Linseman

**Affiliations:** ^1^Research Service, Veterans Affairs Medical CenterDenver, CO, USA; ^2^Department of Biological Sciences and Eleanor Roosevelt Institute, University of DenverDenver, CO, USA; ^3^Division of Clinical Pharmacology and Toxicology, Department of Medicine and Neuroscience Program, University of Colorado DenverAurora, CO, USA

**Keywords:** Rho GTPase, Rac GTPase, neurons, apoptosis, neurodegeneration

## Abstract

The Rho family of GTPases belongs to the Ras superfamily of low molecular weight (∼21 kDa) guanine nucleotide binding proteins. The most extensively studied members are RhoA, Rac1, and Cdc42. In the last few decades, studies have demonstrated that Rho family GTPases are important regulatory molecules that link surface receptors to the organization of the actin and microtubule cytoskeletons. Indeed, Rho GTPases mediate many diverse critical cellular processes, such as gene transcription, cell–cell adhesion, and cell cycle progression. However, Rho GTPases also play an essential role in regulating neuronal morphology. In particular, Rho GTPases regulate dendritic arborization, spine morphogenesis, growth cone development, and axon guidance. In addition, more recent efforts have underscored an important function for Rho GTPases in regulating neuronal survival and death. Interestingly, Rho GTPases can exert either a pro-survival or pro-death signal in neurons depending upon both the cell type and neurotoxic insult involved. This review summarizes key findings delineating the involvement of Rho GTPases and their effectors in the regulation of neuronal survival and death. Collectively, these results suggest that dysregulation of Rho family GTPases may potentially underscore the etiology of some forms of neurodegenerative disease such as amyotrophic lateral sclerosis.

## INTRODUCTION

The Rho GTPase family belongs to the Ras superfamily of low molecular weight (∼21 kDa) guanine nucleotide binding proteins. Although the Rho GTPase family is further divided into seven subfamilies (Rho, Rac, Cdc42, Rnd, RhoD, RhoBTB, and RhoH), the most extensively studied members are RhoA, Rac1, and Cdc42. Multiple studies have demonstrated that Rho family GTPases are important regulatory molecules that link surface receptors to the organization of the actin and microtubule cytoskeletons. In particular, RhoA mediates the formation of stress fibers and focal adhesions, Rac1 induces lamellipodia formation and membrane ruﬄes, and Cdc42 elicits formation of filopodia and microspikes (Hall, 1998). However, the involvement of Rho GTPases in regulating other essential cellular functions has also been described, such as gene transcription (Hill et al., 1995), cell cycle progression (Olson et al., 1995), and cellular survival and death (Heasman and Ridley, 2008; Linseman and Loucks, 2008).

**FIGURE 1:**
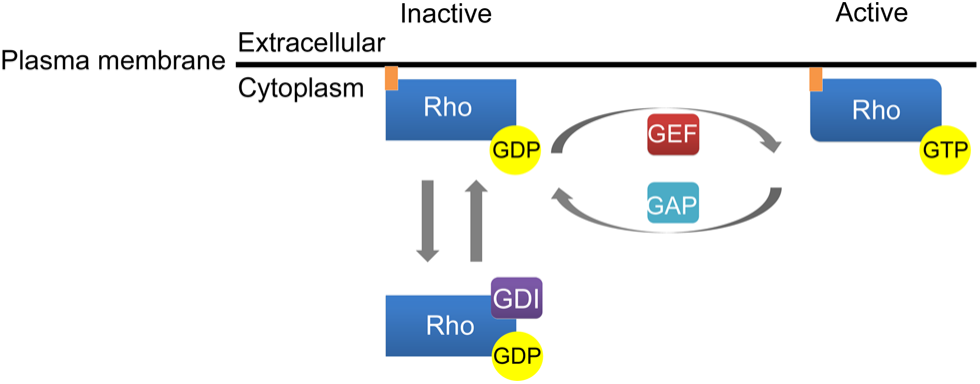
**Activation of Rho family GTPases.** Guanine nucleotide exchange factors (GEFs) catalyze the conversion of GDP to GTP to enhance Rho GTPase activation. GTPase activating proteins (GAPs) inactivate Rho GTPases by stimulating the ability of Rho GTPases to hydrolyze GTP to GDP. Finally, guanine dissociation inhibitors (GDI) regulate Rho GTPase activation by sequestering Rho GTPases in a GDP-bound state in the cytosol.

Similar to Ras family GTPases, activation of Rho GTPases is regulated by cycling between an inactive GDP-bound state and an active GTP-bound state (**Figure 1**). Rho family GTPase activation is facilitated by guanine nucleotide exchange factors (GEFs) that promote the exchange of GDP for GTP while inactivation of Rho GTPases is induced by GTPase activating proteins (GAPs) that stimulate the intrinsic ability of Rho GTPases to hydrolyze GTP to GDP. The third class of molecules that regulate Rho GTPase activity, guanine dissociation inhibitors (GDIs), sequester Rho GTPases in a GDP-bound state in the cytosol. The dissociation of GDIs is required for membrane localization and subsequent activation of Rho family GTPases (**Figure 1**). Rho family GTPases contain an ∼200 amino acid residue Dbl homology (DH) domain and an adjacent, C-terminal, 120 residue pleckstrin homology (PH) domain. The DH domains of Rho GTPases facilitate interaction with GEFs and the subsequent exchange of GTP for GDP while the PH domains bind phosphoinositides to localize Rho GTPases to the plasma membrane (Rossman et al., 2005). More recent studies have elucidated ubiquitin-dependent proteasomal degradation of Rho family GTPases as a novel mechanism controlling their signaling output.

In accordance with a conserved function in regulating cytoskeletal dynamics, Rho GTPases are critical mediators of neuronal growth cone dynamics, dendritic spine formation, and axonal pathfinding. Indeed, an early study highlighted a role for Rho family GTPases in regulating neuronal polarity as cultured hippocampal neurons exposed to either cytochalasin D or *Clostridium difficile* Toxin B (ToxB; an inhibitor of Rho, Rac, and Cdc42) demonstrated a complete loss of F-actin concomitant with the formation of multiple axon-like protrusions, underscoring a role for Rho GTPases in neuronal polarization (Bradke and Dotti, 1999). Additional studies have highlighted that Rac/Cdc42 and Rho typically exhibit an antagonistic relationship to determine neuronal morphology. For instance, an elegant study by Kozma et al. (1997) demonstrated that microinjection of Rac or Cdc42 into N1E-115 neuroblastoma cells enhanced growth cone development and neurite outgrowth, whereas the Rho inhibitory cytotoxin *Clostridium botulinum* C3 coenzyme abolished Rho-dependent growth cone collapse and neurite retraction. In addition to growth cone remodeling, in the regulation of dendritic spine formation, stimulation of *N*-methyl-d-aspartate (NMDA) receptors increases Rac1 and Cdc42 activities while decreasing RhoA activity to promote dendritic growth and branching (Newey et al., 2005). Thus, Rho family GTPases are essential regulators of neuronal morphology.

Given the dynamic nature of the neuronal cytoskeleton, precise spatial and temporal regulation of Rho family GTPases is required for proper neuronal morphology. For example, a previous FRET-based study demonstrated that although PC12 cells exposed to nerve growth factor show enhanced activation of Rac1 and Cdc42 in extending growth cones, Rac1 activation occurs primarily in the distal half of growth cones while Cdc42 activity is localized to the distal microspikes of neuronal growth cones (Aoki et al., 2004). Nonetheless, although tight regulation of Rho GTPases is often attributed to the spatio-temporal activation of GEFs and GAPs, more recent data utilizing novel space- and time-sensitive approaches have begun to unravel complex signaling networks that control the precise activation of Rho family GTPases in neurons and other cells (reviewed in Pertz, 2010). These data demonstrate the critical importance of maintaining precise spatio-temporal regulation of Rho family GTPases.

Similar to the opposing functions exerted between members of the Rho GTPase family in the maintenance of neurite outgrowth and growth cone formation, Rac activation typically promotes neuronal survival while Rho activation often elicits neuronal death (Luo, 2000; Linseman and Loucks, 2008). In the current review, we focus on the involvement of Rac GTPase and Rho GTPase in regulating neuronal survival. We first discuss the critical functions of Rho family GTPases in mediating neuronal survival *in vitro*. Next, we review the essential role that Rho GTPases play in proper nervous system development. Finally, we examine evidence supporting dysregulation of Rho family GTPases as a causative factor in various neurodegenerative diseases.

## Rac GTPase AND Rho GTPase ACT IN AN ANTAGONISTIC MANNER TO REGULATION NEURONAL SURVIVAL

**FIGURE 2:**
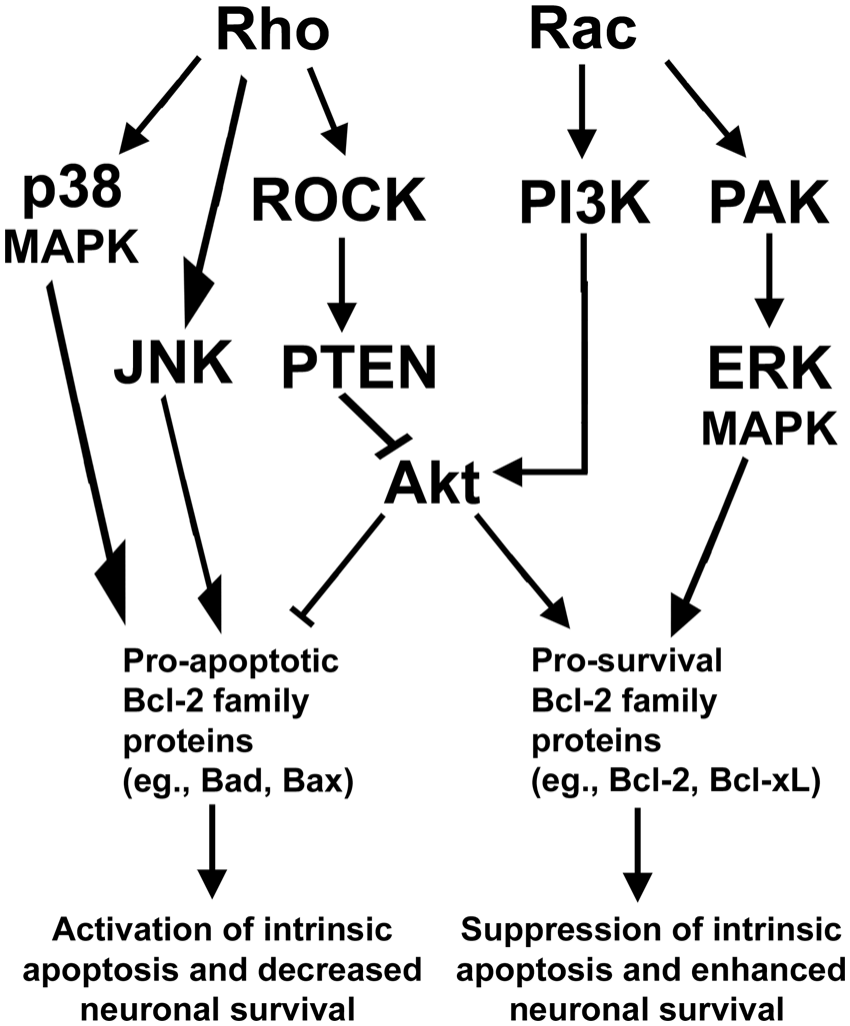
**Rho GTPase signaling pathways regulating neuronal survival.** Rho activates a downstream ROCK/PTEN pathway that indirectly inactivates the pro-survival protein Akt to suppress neuronal survival. Rho can also function to provoke apoptosis via activation of either p38- or JNK-dependent signaling pathways which elicit apoptosis through activation of pro-apoptotic members of the Bcl-2 family of proteins. In opposition to Rho, Rac signals through PI3K to activate Akt to enhance neuronal survival. Rac can also promote neuronal survival via activation of PAK and enhanced ERK-dependent stimulation of pro-survival members of the Bcl-2 family of proteins.

The essential functions of Rho family GTPases in regulating neuronal growth cone formation, neurite outgrowth, and nervous system development suggest that Rho GTPases have an important and conserved function in mediating neuronal survival and death. Indeed, similar to the opposing functions exerted between members of the Rho GTPase family in maintenance of neurite outgrowth and growth cone formation, Rac activation typically promotes neuronal survival while Rho activation elicits neuronal death. In further support of an antagonistic relationship between Rac GTPase and Rho GTPase, recent efforts have identified a key signaling network that maintains neuronal survival downstream of these Rho family GTPases, discussed in detail below (**Figure 2**).

Typically, Rac and its downstream effectors promote neuronal survival, while Rho and its downstream effectors are capable of inducing neuronal apoptosis. In particular, Rac GTPase can signal to the downstream effector p21-activated kinase (PAK) to promote survival of neurons (Johnson and D’Mello, 2005; Loucks et al., 2006). In neurons and other cells, PAK is known to promote cellular survival through activation of mitogen activated protein kinase (MAPK) pathways which inhibit pro-apoptotic members (e.g., Bad) and enhance the expression of pro-survival members (e.g., Bcl-xL) of the Bcl-2 family of proteins (Zha et al., 1996; Schürmann et al., 2000; Loucks et al., 2006; Stankiewicz et al., 2012). In addition, Rac stimulates the key prosurvival phosphatidyl inositol-3 kinase (PI3K)/Akt pathway that inhibits multiple pro-apoptotic Bcl-2 family proteins (e.g., Bad and Bax) while inducing pro-survival Bcl-2 expression. These Rac-dependent pathways actively suppress mitochondrial apoptosis but are antagonized by Rho through activation of its downstream effector, Rho kinase (ROCK), which activates the Akt-inhibitory phosphatase tension homolog (PTEN) phosphatase (**Figure 2**). The inhibition of Akt by the Rho/ROCK/PTEN pathway occurs indirectly as PTEN dephosphorylates phosphatidylinositol (3, 4, 5)-triphosphate (PIP3) into phosphatidylinositol (4,5)-biphosphate (PIP2) to terminate Akt activation (Lai et al., 2014). Maintaining a balance between these Rac dependent pro-survival signals and Rho-dependent pro-apoptotic signals is essential to the survival of many types of neurons including cerebellar granule neurons (CGNs) and spinal motor neurons.

## Rac GTPase AND Rho GTPase REGULATE NEURONAL SURVIVAL AND APOPTOSIS *IN VITRO*

Early studies utilizing 3-hydroxy-3-methylglutaryl-CoA (HMG-CoA) reductase inhibitors (statins) highlighted a likely role for Rho GTPases in regulating neuronal apoptosis. For example, inhibitors of HMG-CoA reductase decrease the localization of Rho GTPases to the plasma membrane by suppressing the synthesis of mevalonate, a precursor required for the isoprenylation of Rho GTPases. Consequently, these compounds induce apoptosis in human brain explants and in a variety of *in vitro* neuronal models, including brain neuroblasts, PC12 cells, and primary rat cortical, hippocampal, and CGNs (Kumano et al., 2000; Tanaka et al., 2000; Garcia-Roman et al., 2001; Meske et al., 2003; März et al., 2007). More recent studies performed *in vitro* in diverse neuronal cell types have clarified that while Rac GTPase typically promotes neuronal survival, Rho GTPase generally provokes neuronal apoptosis.

In recent years, an essential pro-survival function for Rac GTPase has been described in many diverse *in vitro* neuronal models. In particular, we have demonstrated a central pro-survival function for Rac GTPase in CGNs. We have previously shown that *Clostridium difficile* ToxB, a broad-spectrum inhibitor of Rho, Rac, and Cdc42, induces apoptosis of primary CGNs predominantly through deactivation of Rac1 GTPase (Linseman et al., 2001; Le et al., 2005; Loucks et al., 2006). Indeed, in healthy CGNs, Rac GTPase promotes neuronal survival through activation of a pro-survival MEK1/2/ERK1/2 signaling cascade, acting downstream of PAK. This MEK1/2/ERK1/2 signaling cascade functions to repress c-Jun N-terminal kinase (JNK)/c-Jun-dependent induction of the pro-apoptotic BH3-only protein Bim. In addition, it inhibits a pro-apoptotic JAK/STAT5 signaling cascade that suppresses transcription of the pro-survival protein Bcl-xL (Linseman et al., 2001; Le et al., 2005; Loucks et al., 2006; Stankiewicz et al., 2012). These finding demonstrate an essential role for Rac1 in regulating pro-apoptotic members of the BH3-only subfamily of Bcl-2 proteins.

Cerebellar granule neurons are not the only neuronal population that shows dependency on Rac function for its survival. For instance, expression of a dominant-negative mutant of Rac1 in primary cultures of embryonic rat spinal motor neurons is sufficient to induce significant cell death and cause suppression of axon outgrowth in the remaining motor neurons (Jacquier et al., 2006). Moreover, in a motor neuronal cell line, expression of constitutively active Rac1 protects these cells from the deleterious effects of Cu, Zn-superoxide dismutase (SOD1) mutants (Kanekura et al., 2005). Consistent with many other studies, this Rac-dependent neuroprotection was mediated through a P13K/Akt pathways, More recently, cell death induced in primary cultures of rat cerebral cortical neurons by exposure to methylmercury was shown to involve a marked downregulation of Rac1 expression (Fujimura et al., 2009). In a similar manner, primary cultured cortical neurons were induced to undergo apoptosis by expression of dominant-negative Rac1, but were protected from glutamate-induced cell death by expression of constitutively active Rac1 (Johanna et al., 2010).

In opposition to the pro-survival signaling cascades that are activated by Rac GTPase in diverse *in vitro* neuronal models, activation of Rho GTPase typically elicits neuronal apoptosis in response to multiple degenerative insults. For example, RhoA GTPase activation elicits p38alpha/MAPK-dependent CGN apoptosis following glutamate-induced excitotoxicity (Semenova et al., 2007). Interestingly, RhoA-induced apoptosis was sensitive to the pro-survival protein Bcl-2, further highlighting the ability of Rho family GTPases to transmit signals to downstream apoptotic machinery. Similarly, a RhoA-p38alpha MAPK pro-apoptotic pathway is induced by NMDA in primary cortical and hippocampal neurons (Semenova et al., 2007). In an *in vitro* model of Phenylketonuria brain injury, phenylalanine induced mitochondrial-dependent apoptosis of cortical neurons via a mechanism consistent with activation of RhoA. Furthermore, brain-derived neurotrophic factor protected cortical neurons from phenylalanine-induced apoptosis by suppressing Rho activation (Zhang et al., 2010). Finally, corticohippocampal neurons deficient in RhoB are resistant to staurosporine-induced apoptosis (Barberan et al., 2011). Collectively, these data indicate that aberrant activation of RhoA and/or RhoB may contribute to neuronal apoptosis in a variety of *in vitro* models of neurodegeneration.

The pro-apoptotic effects of Rho in neurons are often attributed to its downstream effector, ROCK. In NMDA-induced excitotoxicity in the rat retina, both RhoA and ROCK2 levels are significantly increased prior to neuronal death. Moreover, a ROCK inhibitor, fasudil, prevents the upregulation of both RhoA and ROCK2 and reduces cell loss (Kitaoka et al., 2004). Fasudil also enhances neuronal viability in brain slices or PC12 cells exposed to oxygen-glucose deprivation (Yamashita et al., 2007; Li et al., 2009b). Rho/ROCK signaling does not only act in a pro-death manner downstream of ischemic or excitotoxic stimuli. In addition, a Rho/ROCK pathway also mediates the pro-apoptotic effects of phenylalanine in cultured cerebral cortical neurons (Zhang et al., 2007). Finally, ROCK inhibitors significantly protect cultured cortical neurons from toxicity induced by either aluminum or methylmercury exposure (Chen et al., 2010; Fujimura et al., 2011). Collectively, the above findings indicate that Rho/ROCK signaling often acts in opposition to Rac pro-survival signaling to promote neuronal apoptosis.

Many studies in neurons support the general model that Rac/Cdc42 elicit neurite outgrowth and growth cone development while Rho induces neurite retraction and growth cone collapse. However, it is important to note that, in certain circumstances, Rho family GTPases can have unanticipated effects on neuronal morphology. For example, expression of a dominant negative mutant of Rac1 has been demonstrated to promote neurite outgrowth in dorsal root ganglion (DRG) cells (Fournier et al., 2003) and constitutively active Rac1 increases the proportion of collapsed growth cones in DRG neurons (Jin and Strittmatter, 1997; Vastrik et al., 1999). Similarly, constitutive activation of Rac1 was shown to decrease the length of the longest neurite in mouse cortical neurons (Kubo et al., 2002). Furthermore, in some studies, Rho GTPase has been demonstrated to induce neurite formation. Indeed, expression of dominant negative RhoA inhibited axon growth in cultured hippocampal neurons (Ahnert-Hilger et al., 2004). Moreover, it is interesting to note that exposure of CGNs to low concentrations of the neural chemokine stromal cell-derived factor-1 (SDF-1) elicited RhoA-dependent axonal elongation, while CGNs subjected to high concentrations of SDF-1 demonstrated inhibition of axon formation (Arakawa et al., 2003). While these apparent discrepancies in Rho family GTPase functions may reflect differences in either organism specific- or cell type-specific regulation of neuronal morphology, they also further underscore the importance of tight regulation of Rho GTPase activation in order for proper nervous system development.

## Rho GTPases IN NERVOUS SYSTEM DEVELOPMENT

In the nervous system, Rho family GTPases play major and evolutionarily conserved roles in the development, migration, and plasticity of neurons. Rho, Rac, and Cdc42 have all been shown to regulate dendritic growth and arborization, as well as axon guidance (Threadgill et al., 1997; Li et al., 2002; Yuan et al., 2003). In contrast to the induction of neurite outgrowth, axonal extension, and dendritic spine morphogenesis typically elicited by Rac, Rho generally opposes these effects in the central nervous system (CNS; Linseman and Loucks, 2008). Here, we will briefly review the involvement of Rac and Rho in neuronal development.

### Rac IN NEURONAL DEVELOPMENT

The three mammalian Rac isoforms that exist (Rac1, Rac2, and Rac3) are ∼90% homologous (Didsbury et al., 1989; Haataja et al., 1997). However, the differential expression patterns of these three isoforms of Rac indicate that they may have tissue-specific functions during nervous system development. While Rac1 is ubiquitously expressed, Rac2 is mainly expressed in the hematopoietic system, and Rac3 is expressed in the brain. Intriguingly, Rac1 is expressed diffusely in the murine brain during development, while Rac3 expression peaks during times of neuronal maturation and synaptogenesis (Bolis et al., 2003). Rac1-deficient mice fail to complete gastrulation and are embryonic lethal at E8.5, demonstrating defects in cell adhesion and migration (Sugihara et al., 1998). Although Rac2- and Rac3-deficient mice are viable and fertile, certain strains of these knockouts display signs of cell type-specific defects in the hematopoietic system and brain, respectively (Pedersen and Brakebusch, 2012). Here, we will focus on the function of Rac1 and Rac3 in regulating neuronal development.

While loss of function mutations in *Drosophila* Rac genes result in defects in axonal growth, guidance, and branching (Hakeda-Suzuki et al., 2002; Ng et al., 2002), the involvement of Rac in vertebrate nervous system development is less clear. Given that genetic inactivation of Rac1 results in embryonic lethality in mice, more recent studies have focused on the effects of tissue-specific deletion of Rac1 on nervous system development, confirming an important role for Rac1 in neurite outgrowth and axonal pathfinding, as well as neuronal migration. Indeed, conditional deletion of Rac1 in whole murine brain increased apoptosis and impaired axonal pathfinding and neuronal migration in CGNs (Tahirovic et al., 2010). Consistent with a critical role for Rac1 in axon guidance during development of the vertebrate CNS, restricted deletion of Rac1 in the ventral telencephalon of mice prevents axonal migration across the midline of the corpus callosal and hippocampal commissures but does not affect axonal outgrowth, indicating that Rac1 primarily controls axon guidance rather than outgrowth in these brain regions (Chen et al., 2007). Nonetheless, Rac1 regulates mammalian neuronal development in a cell type-specific manner as the Rac-specific GEF, Tiam1, has been shown to enhance both neurite outgrowth and axon formation via its activation of Rac1 (Kunda et al., 2001).

More recent studies have uncovered an important function for Rac1 GTPase in neuronal proliferation. For example, deletion of Rac1 in the neural crest enhanced cell-cycle exit and diminished both the self-renewal and proliferation of late stage neural crest-derived stem cells (Fuchs et al., 2009). However, genetic deletion and virus-based loss of function studies demonstrated that Rac1 is not required for the proliferation of neural stem cell/progenitor cells (NS) in the hippocampus during embryonic development, but is an important regulator of late dendritic growth and spine maturation (Vadodaria et al., 2013). Nonetheless, the involvement of Rac1 in mediating proliferation in additional brain regions has also been suggested in recent literature. For instance, in the murine forebrain, loss of Rac1 reduced proliferation while increasing cell-cycle exit and premature differentiation of progenitor cells in the subventricular zone (Leone et al., 2010). Interestingly, a novel function was recently described for Rac1 in mediating learning-evoked neurogenesis in the hippocampus of adult mice. While previous reports have demonstrated that increased production of adult-born neurons in the hippocampus occurs in a Rac1-independent manner *leading up* to learning events, Haditsch et al. (2013) revealed that neuronal loss of active Rac1 attenuated proliferation and accumulation of neural progenitors *during* learning events. Future work will be required to further elucidate the involvement of Rac1 in mediating neural progenitor proliferation during nervous system development.

While *in vitro* and *in vivo* evidence suggests a crucial function for Rac1 in nervous system development, to date, relatively few studies have highlighted a similar function for Rac3. For example, genetic disruption of Rac3 in mice did not lead to obvious developmental defects and histopathological and immunohistochemical analysis did not reveal any abnormalities in the gross morphology of Rac3^-/-^ brains when compared to wild type mice. However, Rac3 knockout mice learned to perform significantly better on a rotarod test than wild type mice, indicating that loss of Rac3 improves motor learning and retention of coordinated memory behaviors (Corbetta et al., 2005). Rac3 may perform overlapping functions with Rac1 during nervous system development as neuronal-specific knockout of both Rac1 and Rac3 in mice led to behavioral defects, epilepsy, and premature death at postnatal day 9. Further analysis of brain tissue in Rac1^-/-^/Rac3^-/-^ mice revealed developmental abnormalities in the hippocampus when compared to wild type brains (Corbetta et al., 2009). Thus, evidence suggests that both Rac1 and Rac3 are important for proper CNS development.

### Rho IN NEURONAL DEVELOPMENT

While three different isoforms of Rho (RhoA, RhoB, RhoC) have been identified, the involvement of RhoA in nervous system development is best characterized. Nonetheless, RhoB and RhoC share many effectors with RhoA, suggesting potentially overlapping functions. However, while RhoA and RhoC localize to the cell membrane, RhoB localizes to the membranes of intracellular vesicles. Furthermore, RhoB and RhoC knockout mice do not display any obvious developmental defects while RhoA knockout mice are embryonic lethal (Pedersen and Brakebusch, 2012). The involvement of RhoA during development has been further elucidated through tissue-specific deletion of RhoA in the CNS.

Consistent with a role in regulating cytoskeletal dynamics, deletion of RhoA in spinal cord neuroepithelium, midbrain, and forebrain each led to loss of both adherens junctions and apical-basal polarity (Herzog et al., 2011; Katayama et al., 2011). Deletion of RhoA in neuroepithelium resulted in embryonic lethality and abnormalities in the organization of the ventricular region (Herzog et al., 2011). While deletion of RhoA in neuroepithelium resulted in decreased proliferation of neural progenitors, deletion of RhoA from the midbrain led to increased proliferation of neural progenitor cells (Katayama et al., 2011). Thus, it is likely that RhoA exerts cell type-specific functions during development of the nervous system.

Using a gene knock-in strategy in which mice express dominant-negative RhoA in all developing neurons, Sanno et al. (2010) showed that suppression of Rho activity results in significant increases in the density and absolute number of neurons in the somatosensory cortex compared to wild type littermates, and this effect is due to decreased neuronal apoptosis during early postnatal development. In neural progenitors, it also appears that RhoA may mediate survival as expression of a dominant negative mutant of RhoA enhanced survival and proliferation of NSPCs (Numano et al., 2009). Thus, recent studies have uncovered RhoA as an important regulator of neuronal survival and neural stem cell proliferation.

In a similar manner to Rac1 GTPase, targeted deletion of RhoA in the cerebral cortex of mice revealed a role for RhoA in regulating neuronal migration. Mice lacking RhoA in the cerebral cortex displayed both subcortical band heterotopias and cobblestone lissencephaly, both of which are attributed to defects in neuronal migration. While RhoA^-/-^ neurons migrated normally when transplanted into wild type cerebral cortex, wild type neurons did not migrate properly when transplanted into RhoA^-/-^ cerebral cortex. Intriguingly, the defects in neuronal migration were not due to diminished RhoA activity in neurons, but were attributed to destabilization of the actin and microtubule cytoskeleton of the radial glia scaffold, which directs migrating neurons (Cappello et al., 2012). Therefore, unlike the role of Rac1 in regulating neuronal migration, limited evidence suggests that RhoA may not be required for migration in neurons but instead directs migration through its effects on the actin and microtubule cytoskeleton in radial glia cells.

While the involvement of RhoA in neuronal development has been a recent subject of investigation, relatively few studies have examined the role of RhoB in CNS development. Nonetheless, RhoB^-/-^ mice have been generated and while loss of RhoB does not result in overt signs of developmental defects, mice lacking RhoB do display signs of memory impairment as paired-pulse facilitation, post-tetanic potentiation, and the early phase of long-term potentiation (LTP) are all reduced in RhoB-null mice. Consistent with the essential role dendritic spines morphogenesis in LTP, spine number was decreased and dendritic branching was increased in hippocampal neurons of RhoB^-/-^ mice (McNair et al., 2010). Thus, recent studies have highlighted functions for both RhoA and RhoB in proper nervous system development.

## DYSREGULATION OF Rac GTPase AND Rho GTPase IN NEURODEGENERATIVE DISEASES AND NEURONAL INJURY

The essential functions of Rac GTPase and Rho GTPase in mediating nervous system development and neuronal survival indicate that dysregulation of these GTPases may play a causative role in the pathology of some forms of neurodegenerative disease. Indeed, dysregulation of Rac and/or Rho GTPase has been reported in a variety of neurodegenerative diseases and neuronal traumas, including amyotrophic lateral sclerosis (ALS), Alzheimer’s disease (AD), Parkinson’s disease (PD), ischemia/reperfusion, amongst many others. Here, we will review recent advances examining dysregulation of these GTPases in various models of neurodegenerative disease (**Table 1**).

**Table 1:** Dysregulated Rho family GTPase signaling in neurodegenerative disease.

Protein	Neurodegenerative disease
Rac (GTPase)	ALS: siRNA knock down or inhibition of Rac induces death of NSC34 cells and primary motor neurons (Kanekura et al., 2005; Jacquier et al., 2006); activation of Rac in microglia induces death of neighboring motor neurons (Harraz et al., 2008; Apolloni et al., 2013); G93A mSOD1 decreases Rac activity in SH-SY5Y cells and increases Rac1 activity in microglia (Pesaresi et al., 2011; Apolloni et al., 2013) AD: diminished expression in patient brains (Zhao et al., 2006); activates APP transcription (Wang et al., 2009); positive regulation of Aβ production (Boo et al., 2008; Wang et al., 2009); Aβ activated Tiam1/Rac activation (Mendoza-Naranjo et al., 2007, 2012); human neuroblastoma cells exposed to Aβ to showed diminished activation of Rac1 (Petratos et al., 2008) HD: interacts with mutant Htt and knockdown of Rac1 reduces caspase-3/7 activity in striatal cells expressing mutant Htt (Tourette et al., 2014) PD: wild type LRRK2 activates Rac1 while mutant LRRK2 does not, overexpression of Rac1 rescues SH-SY5Y cells from mutant LRRK2 (Chan et al., 2011); inhibition of Rac decreased rotenone-induced ROS production from microglia (Zhou et al., 2012); inhibition of Rac1 reduced 6-OHDA-induced oxidative damage in dopaminergic cells (Philippens et al., 2013) SCI: Rac1 mediates myelin fragmentation (Jung et al., 2011); Rac1 improved survival and axonal regeneration in optic nerve crush (Lorenzetto et al., 2013) I/R: activity is increased (Johanna et al., 2010) or decreased (Zhang et al., 2009); total Rac1 localizes to degenerating neurites (Johanna et al., 2010); may increase production of ROS (Raz et al., 2010)
PAK (Rac effector)	AD: decreased localization in hippocampal sections but increased intraneural aggregates in patients (Zhao et al., 2006); translocated from the cytosol to granule intracellular bodies that also stain positive for Rac and Aβ (Ma et al., 2008); increased activity following Aβ42 exposure in hippocampal neurons (Mendoza-Naranjo et al., 2012) HD: PAK1 and PAK2 bind to mutant Htt, PAK1 colocalizes with mutant Htt in human brains (Luo et al., 2008)
Alsin (Rac GEF)	Juvenile-onset ALS: loss of function mutations (Yamanaka et al., 2003; Hadano et al., 2007)
ARFGEF16 (Rac GEF)	Sporadic ALS: hypermethylated and downregulated in patients (Figueroa-Romero et al., 2012)
RNGEF (Rho GEF)	ALS: forms cytoplasmic inclusions in sporadic and familial patients (Keller et al., 2012; Droppelmann et al., 2013)
ROCK (Rho effector)	ALS: ROCK activity is increased in G93A mSOD1 mice and patients (Hu et al., 2003; Capitanio et al., 2012; Conti et al., 2014); inhibition of ROCK delays onset and extends survival in the G93A mSOD1 mouse (Takata et al., 2013; Tönges et al., 2014) AD: Rho/ROCK increased production of toxic Aβ fragments (Zhou et al., 2003) HD: inhibition of ROCK improves rotarod performance and reduces soluble mutant Htt in R6/2 mouse (Li et al., 2009a) PD: inhibition of ROCK delays onset and extends survival in mice administrated MPTP (Tönges et al., 2012); ROCK inhibition prevented microglia from eliminating dopaminergic neurons in MPTP-treated mice (Barcia et al., 2012) SCI: inhibition or knockout of ROCK is protective (Fournier et al., 2003; Duffy et al., 2009; Wu et al., 2009) I/R: ROCK increased threefold in ischemic mice (Shin et al., 2008); localizes to actin neurofilaments (Yamashita et al., 2007), fasudil protects in rodent models (Rikitake et al., 2005; Satoh et al., 2007; Wei et al., 2014)
∝-pix (Rac GEF)	HD: promotes mutant Htt aggregation in MG251 cells (Eriguchi et al., 2010)
Kalirin-7 (Rac GEF)	AD: Kalirin-7 mRNA and protein levels are decreased in human AD hippocampal samples (Youn et al., 2007)
Rho (GTPase)	AD: RhoA is decreased in human AD brains and mice overexpressing Swedish mutant APP, RhoA increased in degenerating neurites of Swedish Aβ mutant mice (Huesa et al., 2010); human neuroblastoma cells exposed to Aβ show enhanced activation of RhoA and diminished activation of Rac1 (Petratos et al., 2008) SCI: inhibition of Rho protects (Boato et al., 2010; Boomkamp et al., 2012) I/R: Rho is upregulated following stroke in humans (Brabeck et al., 2003) and mice (Trapp et al., 2001; Erdo et al., 2004)
ARHGEF10 (Rho GEF)	CMTD: constitutively active in patients with slowed nerve conduction velocities and thin myelination of peripheral nerves (Verhoeven et al., 2003; Chaya et al., 2011)

### AMYOTROPHIC LATERAL SCLEROSIS

Amyotrophic lateral sclerosis is a devastating neuromuscular disorder characterized by loss of motor neurons of the motor cortex, brain stem, and upper and lower spinal cord. Approximately 10% of ALS cases appear to be caused by a genetic component, while the remaining 90% are considered sporadic in nature. In 1993, the glycine to alanine substitution at position 93 in Cu, Zn- superoxide dismutase (SOD1) was one of eleven mutations initially identified to be causative in familial ALS. Although the G93A SOD1 mutation remains the best characterized to date, mutations in additional genes such as Alsin (ALS2), Tar DNA-binding protein 43 (TDP43), and fused in sarcoma (FUS) have also been linked to other familial forms of ALS. Recent evidence suggests that dysregulation of Rho GTPases may be a factor underlying both genetic and sporadic ALS.

Recessive mutations in a Rac GEF, alsin, have been reported in juvenile forms of ALS, primary lateral sclerosis, and infantile-onset ascending hereditary spastic paralysis (Hadano et al., 2007). When overexpressed in cultured human cells, alsin mutants are rapidly degraded, suggesting disease progression occurs through a mechanism consistent with alsin loss-of-function (Yamanaka et al., 2003). Although alsin can function as a GEF for both Rab5 and Rac1, alsin colocalizes with Rac1 in the growth cones of neurons and regulates Rac1 signaling and neurite outgrowth through its intrinsic GEF activity, thus suggesting a link between Rac1 disruption and neurodegenerative disease (Topp et al., 2004; Kanekura et al., 2005; Tudor et al., 2005). These studies highlight that a loss of Rac activity may underlie the progression of early onset forms of ALS.

The critical role of Rac in maintaining neuronal survival is evidenced by the fact that disruptions in Rac signaling have been shown to induce apoptosis in a variety of diverse neuronal cell types, including primary motor neurons (Kanekura et al., 2005; Jacquier et al., 2006; Loucks et al., 2006; Stankiewicz et al., 2012). In spinal motor neurons isolated from embryonic rats, siRNA knockdown of alsin induces neurite retraction and cell death that is abrogated by expression of constitutively active Rac1 (Jacquier et al., 2006). Furthermore, alsin antagonizes NSC34 motor neuronal death induced by the expression of ALS-causing SOD1 mutants (mSOD1) via activation of a pro-survival Rac1/PI3K/Akt signaling cascade (Kanekura et al., 2005). Intriguingly, accumulating evidence suggests that SOD1 may regulate the activity of Rac1 GTPase. In particular, neuronal apoptosis induced via expression of either G93A or H80R mSOD1 in SH-SY5Y dopaminergic cells correlated with a decrease in Rac1 GTPase activity. Indeed, expression of a constitutively active Rac1 GTPase mutant abrogated mSOD1-induced neuronal death while expression of dominant negative Rac1 GTPase alone induced apoptosis (Pesaresi et al., 2011). Future studies should be aimed at determining whether expression of mutant forms of SOD1 similarly decrease Rac1 activation in motor neurons in particular.

In addition to mutations that code for alsin and SOD1, recent evidence also suggests that ARHGEF16, a GEF for Rac GTPase, is hypermethylated and downregulated in postmortem sporadic ALS spinal cord samples when compared to samples from neurologically normal controls (Figueroa-Romero et al., 2012). Thus, dysregulation of Rac GTPase may be a common factor underlying both familial and sporadic forms of ALS. While accumulating evidence has highlighted a critical function for Rac GTPase in maintaining neuronal survival, the involvement of Rac in the etiology of ALS has only been examined recently and the exact mechanism by which disruptions in Rac activity may induce neuronal apoptosis remains poorly understood. Therefore, alterations in Rac activity in motor neurons and mouse models of ALS warrants further investigation.

In a manner dissimilar to motor neurons, several studies indicate that mutant forms of SOD1 associated with the pathology of ALS have an opposing function in microglia, ultimately resulting in enhanced activation of Rac1 GTPase in a redox-sensitive manner. For example, Harraz et al. (2008) demonstrated that wild type SOD1 binds to Rac1 and inhibits its intrinsic GTPase activity under reducing conditions, ultimately increasing active Rac1 signaling. In the presence of H_2_O_2_, the interaction between wild type SOD1 and Rac1 GTPase was uncoupled. Intriguingly, ALS causing mutations in SOD1 prevent the enzyme from redox uncoupling, resulting in enhanced and persistent activation of Rac1 GTPase in microglia cells. Inevitably, aberrant Rac1 GTPase activity leads to increased activation of Nox2, a catalytic subunit of NADPH oxidase, resulting in an overproduction of damaging ROS. In agreement with Rac1-dependent activation of NADPH oxidase as a contributing factor underlying motor neuron degeneration in the G93A mSOD1 mouse, inhibition of, or genetic deletion, of Nox2 slows disease progression and increases survival in this particular mouse model of familial ALS (Wu et al., 2006; Marden et al., 2007; Valdmanis et al., 2008). Furthermore, Apolloni et al. (2013) recently identified extracellular ATP as a potential mechanism by which Rac1 GTPase activity may be enhanced in microglia of G93A mSOD1 mice. In primary microglia cells isolated from G93A mSOD1 mice, stimulation of the P2X_7_ receptor by its agonist, 2′-3′-*O*-(benzoyl–benzoyl) ATP, enhanced Nox2 activation and the production of ROS in a Rac1-dependent manner when compared to microglia derived from non-transgenic animals. Thus, recent experimental evidence suggests that G93A mSOD1 may stimulate Rac1 GTPase activation in a P2X_7_-dependent manner to enhance the release of toxic ROS from microglia.

Recent studies also implicate aberrant Rho GTPase activity as an underlying factor in the pathology of ALS. For example, although the significance remains unknown, the Rho GEF, RGNEF, displays enhanced cytoplasmic inclusions in spinal motor neurons from both sporadic and familial ALS patients (Keller et al., 2012; Droppelmann et al., 2013). However, future studies will be required to identify the importance of RGNEF-positive spinal motor neuron cytoplasmic inclusions. Nonetheless, in accordance with enhanced Rho GTPase activity as a causative factor to motor neuronal death in ALS disease progression, ROCK activity is increased in the G93A mSOD1 mouse model of ALS, as well as sporadic ALS patients (Hu et al., 2003; Capitanio et al., 2012; Conti et al., 2014). Tönges et al. (2014) demonstrated that inhibition of ROCK preserved neuromuscular junctions and extended the survival of G93A mSOD1 mice via a mechanism consistent with reduced microgliosis and decreased release of pro-inflammatory cytokines and chemokines such as TNFα and IL-6. When examining motor neuron death in G93A mSOD1 mice, Takata et al. (2013) demonstrated that administration of fasudil slowed disease progression and reduced motor neuron loss by a mechanism consistent with reduced activation of ROCK and PTEN and restored activation of the pro-survival kinase Akt. While the above studies indicate that a gain of Rho GTPase activity may underlie the progression of ALS, new studies will need to be carried out to determine the precise mechanism by which unopposed activation of Rho GTPase may contribute to the motor neuronal death observed in ALS.

### ALZHEIMER’S DISEASE

Alzheimer’s disease is the most common form of dementia and is characterized by extensive loss of neurons in the hippocampus and cerebral cortex. The onset of AD correlates with an aberrant accumulation of extracellular amyloid-β (Aβ) plaques which are the products of proteolytic cleavage of the membrane protein amyloid precursor protein (APP). Aβ plaques have been demonstrated to be at least partially responsible for the dysregulation of actin polymerization that underlies synaptic dysfunction observed during AD progression (Bloom, 2014). Accumulating evidence suggests that dysregulated Rho family GTPase activity may correlate with Aβ production and thereby contribute to the loss of actin polymerization and dendritic spines, which underlies the pathogenesis of AD.

Although diminished Rac1 expression is observed in AD brains obtained from patients early in disease (Zhao et al., 2006), recent evidence suggests a positive correlation between Rac GTPase activity and APP expression during later stages of AD disease progression. For example, in primary hippocampal cells, inhibition of Rac utilizing NSC23766 or expression of a dominant negative Rac1 mutant reduced APP mRNA and APP protein expression, indicating that Rac1 activates APP transcription (Wang et al., 2009). In addition, Rac1 may also contribute to the formation of toxic Aβ fragments as inhibition of Rac1 diminished gamma secretase-dependent cleavage of APP in COS-7 cells (Boo et al., 2008). Interestingly, while these data highlight that Rac1 may contribute to APP transcription and the generation of toxic Aβ fragments, recent reports also suggest that Aβ peptides can in turn, induce the activation of Rac1. For example, addition of Aβ42 to hippocampal cultures resulted in upregulation of the Rac-specific GEF Tiam1, enhanced activation of Rac1, downstream activation of the Rac effectors PAK1 and LIMK, and increased actin polymerization (Mendoza-Naranjo et al., 2007, 2012). Thus, Rac1 GTPase may function in a positive feedback loop with APP and/or Aβ fragments to regulate the expression and activities of one another.

Further evidence suggests that alterations of a Rac1-dependent signaling cascade links expression of toxic Aβ fragments to disruption of actin polymerization and dendritic spines. Indeed, it has previously been reported that expression of the downstream Rac effector, PAK, is decreased in hippocampal sections, while levels of phosphorylated (active) PAK were elevated in intraneural aggregates during AD progression (Zhao et al., 2006). In human AD and Tg2576 mice, phosphorylated (active) PAK translocated from the cytosol to intracellular granule bodies that also stain positive for Rac1 and Aβ. In primary hippocampal cells, expression of Aβ induced a similar redistribution of PAK and resulted in a reduction in dendrites (Ma et al., 2008). Therefore, dysregulation of PAK signaling may underlie some synaptic defects observed in AD.

Although these previous studies indicate that enhanced Rac1 activity may contribute to AD progression, the global reduction of Rac activity in the brains of human AD patients reveals that the involvement of Rac GTPase in the progression of AD is complex. In accordance with reduced Rac GTPase activity, it was previously reported that the mRNA and protein expression levels of the Rac GEF, Kalirin-7, are reduced in the hippocampus of human AD brains. Kalirin-7 is known to play a critical role in dendritic spine maintenance. Intriguingly, Kalirin-7 associates with iNOS to downregulate its activity in AtT-20, SH-SY5Y, C6, and Neuro-2A cells. In the human AD hippocampus, elevated expression of iNOS correlates with diminished association with Kalirin-7 (Youn et al., 2007). Thus, these data suggest that reduced Kalirin-7 levels may contribute to the pathogenesis of AD through enhanced activity of iNOS. Future studies will be critical in determining whether reduced levels of Kalirin-7 contribute to diminished Rac GTPase activity during early stages of AD progression.

In addition to Rac GTPase, aberrant Rho GTPase activity has also been linked to the pathology of AD. Indeed, RhoA is decreased in human AD brains and in the brains of transgenic mice overexpressing the Swedish double mutant of APP. Moreover, while RhoA was decreased in synapses of AD mice, its expression was increased in degenerating neurites consistent with the involvement of RhoA in neurite retraction (Huesa et al., 2010). Addition of Aβ to human neuroblastoma cells resulted in reduced neurite length concomitant with enhanced activation of RhoA and diminished activation of Rac1 (Petratos et al., 2008). Activation of RhoA has also been correlated with production of Aβ fragments as activation of Rho and its downstream effector, ROCK, increased production of toxic Aβ fragments by gamma secretase-dependent cleavage of APP (Zhou et al., 2003). Thus, aberrant activation of Rho GTPase may contribute to AD pathology through enhanced neurite retraction, as well as, increased production of toxic Aβ fragments.

### HUNTINGTON’S DISEASE (HD)

Huntington’s disease (HD) is a progressive and fatal autosomal dominant disorder characterized by loss of cells in the striatum and cortex, resulting in involuntary body movements (chorea), cognitive decline, psychiatric conditions, and eventually death. HD is caused by a mutation in the *Huntingtin* gene which results in expansion of a CAG nucleotide repeat. Mutant huntington (Htt) forms toxic gain-of-function aggregates that underlie the etiology of HD. Although relatively few studies have examined the involvement of Rho GTPases in the progression of HD, recent efforts have linked dysregulated Rho family GTPase signaling to both the production of toxic ROS and mutant Htt aggregation in the degenerating CNS.

In a similar manner to other neurodegenerative diseases, the production of ROS is believed to play a role in the etiology of HD. Compared to neurologically normal controls, HD patients show enhanced NADPH oxidase activity and an increase in ROS in the cortex and striatum of postmortem brain samples. In the 140Q/140Q mouse model of HD, NADPH oxidase activity in cortex and striatum correlated with enhanced production of ROS and neurite swellings, and was prevented by treatment with NOX inhibitors. Similarly, mutant HD mice bred with NOX2 knockout mice showed enhanced survival concomitant with decreased NADPH oxidase activity and lowered production of ROS (Valencia et al., 2013). Given the important involvement of Rac1 GTPase in activation of NADPH oxidase activity, this study indicates that aberrant Rac1 activation may contribute to production of ROS and thereby the pathology of HD. In addition to the role of Rac1 in contributing to the production of ROS through activation of NADPH oxidase, Rac1 interacts with mutant Htt in a yeast two-hybrid screen and siRNA-mediated knockdown of Rac1 reduces caspase-3/7 activity in striatal cells expressing mutant Htt (Tourette et al., 2014). Thus, enhanced Rac1 activation may contribute to the pathogenesis of HD through either activation of NADPH oxidase in microglia or direct binding to mutant Htt in susceptible neuronal populations.

The downstream Rac effector, PAK, has also been implicated in the pathogenesis of HD. PAK1 binds to Htt *in vitro* and *in vivo* and colocalizes with mutant Htt in human HD brains. Overexpression of PAK1 enhances the aggregation of mutant Htt and Htt-induced toxicity of HeLa cells independent of its kinase activity (Luo et al., 2008). In addition to PAK1, a recent study utilizing a yeast two-hybrid screen identified PAK2 as a modulator of Htt-induced toxicity (Tourette et al., 2014). Finally, the PAK-interacting exchange factor (∝-pix), a GEF for Rac and Cdc42, promotes mutant Htt aggregation in the MG251 neuronal glioblastoma cell line (Eriguchi et al., 2010). Thus, Rac and its interacting proteins may underlie the neurotoxicity and aggregation of mutant Htt in HD.

Intriguingly, recent studies have also highlighted an important role for ROCK activation in the etiology of HD. Inhibition of ROCK utilizing Y-27632 has been demonstrated to have neuroprotective effects *in vitro* and in a *Drosophila* model of HD. More recently, Y-27632 has also been shown to improve rotarod performance and reduce soluble mutant Htt levels in the R6/2 mouse model of HD. However, inhibition of ROCK did not have a significant effect on brain weight, inclusion number or size, striatal medium spiny neuron number, clasping behavior, or lifespan (Li et al., 2009a). ROCK may underlie HD progression through the phosphorylation and inhibition of profilin, which when overexpressed, reduces the aggregation of polyglutamine-expanded Htt and androgen receptors in primary neurons (Shao et al., 2008). These data indicate that enhanced activation of Rho/ROCK signaling may also contribute to the pathogenesis of HD.

### PARKINSON’S DISEASE

Parkinson’s disease is the most common neurodegenerative movement disorder and is caused by progressive degeneration of nigrostriatal dopaminergic neurons of the midbrain. Clinically, PD manifests as a progressive locomotor, cognitive, and behavioral disorder. In the CNS, PD results in the formation of Lewy bodies containing accumulation of ∝-synuclein. The most common genetic cause of familial PD is due to mutation in leucine-rich repeat kinase 2 (LRRK2; Macleod et al., 2006). Although several genetic mutations have been identified in PD, exposure to environmental toxins has also been strongly associated with development of PD.

Expression of disease-causing LRRK2 mutants induces neurite retraction and dopaminergic apoptotic cell death in primary neurons and in the rodent CNS (Macleod et al., 2006). Interestingly, wild type LRRK2 interacts with and increases the activity of Rac1 *in vitro*; however, the disease-causing mutants, G2019S and R1441C display weakened Rac1 binding while the Y1699C and I2020T mutations confer increased binding of Rac1. Thus, the distinct LRRK2 mutations exert differential effects on Rac1 binding. Consistent with diminished Rac1 activity playing a role in PD pathology, expression of Rac1 rescues SH-SY5Y cells from neurite retraction and cell death caused by disease-causing LRRK2 PD mutants (Chan et al., 2011). Therefore, mutations in LRRK2 may induce neurite retraction and cell death in dopaminergic neurons principally through diminished Rac1 GTPase activity.

Rotenone is an environmental toxin that induces dopaminergic loss and parkinsonian features when chronically administered to rodents (Betarbet et al., 2000; Alam and Schmidt, 2002). It has been previously reported that rotenone induces the production of ROS not only by inhibition complex I, but also through the activation of NADPH oxidase (Gao et al., 2002, 2003). In phagocytes, rotenone directly interacted with the catalytic subunit of NADPH oxidase, NOX2, to enhance activation of NADPH oxidase and the formation of ROS. Rac1 enhanced the activation of rotenone-dependent ROS formation and inhibition of Rac1 utilizing NSC23766 decreased ROS release from microglia (Zhou et al., 2012). In a similar manner, inhibition of NADPH oxidase utilizing apocynin relieved PD symptoms in a marmoset model of the disease (Philippens et al., 2013). These data indicate that aberrant activation of Rac in microglia may contribute to enhanced production of ROS and underlie the death of neighboring dopaminergic neurons in PD.

However, the involvement of Rac in mediating neuronal survival may be much more complex in PD progression as it has also been demonstrated that hippocampal and dopaminergic neurons exposed to rotenone showed decreased active Rac1-GTP and enhanced RhoA-GTP (Sanchez et al., 2008). Thus, in a manner similar to the antagonistic relationship between Rac and Rho in regulating the survival of motor neurons, *diminished* Rac activity and *enhanced* Rho activity may contribute to neuronal death during the progression of some forms of PD, particularly those forms that are linked to environmental toxin exposure. Further, Rac1 may contribute to PD disease progression in a cell type-specific manner, where *loss* of Rac GTPase activity may contribute to the death of neurons while *increased* Rac-GTP activity in microglia may contribute to the formation of toxic ROS.

In addition to rotenone, the neurotoxic 1-Methyl-4-phenyl-1,2,3,6-tetrahydropyridine (MPTP) is taken up by dopaminergic neurons and induces PD symptoms through inhibition of mitochondrial complex I of the electron transport chain, leading to enhanced ROS production and neuronal apoptosis. When administered to mice, MPTP induces cellular polarization of microglia and leads to the formation of neuron-glia contacts, which precede the phagocytosis of dopaminergic cell bodies. This process may be mediated by ROCK activation as inhibition of ROCK utilizing fasudil prevented microglia motility and activation of microglia in the substantia nigra of MPTP-treated mice (Barcia et al., 2012). Indeed, inhibition of ROCK has shown to enhance survival of dopaminergic neurons and attenuate axonal loss in the MPTP mouse model of PD (Tönges et al., 2012). These data indicate that dysregulation of Rho family GTPases may underlie both genetic and environmental cases of PD.

### CHARCOT MARIE-TOOTH DISEASE (CMTD)

Charcot marie-tooth disease (CMTD) is an inherited neurological disorder that presents as progressive muscle weakness and decreased muscle size. The first subtype of CMTD is characterized by demyelination and results in lower nerve conduction velocities while the second subtype is due to a defect within the axons themselves. Recent studies have highlighted that dysregulated Rho family GTPase activity may underlie demyelination and axonal abnormalities. Recently, the T332I mutation was identified in ARHGEF10 in patients exhibiting slowed nerve conduction velocities and thin myelination of peripheral nerves. ARHGEF10 is a GEF for RhoA that is highly expressed in the peripheral nervous system and exhibits enhanced activity when harboring the T332I mutation (Verhoeven et al., 2003; Chaya et al., 2011). Consistent with activation of RhoA, T332I ARHGEF10 induces cell contraction through Rho/ROCK signaling in Schwann cells from rat sciatic nerve. Thus, ARHGEF10 may contribute to the pathology of CMTD by enhanced activation of RhoA-dependent cell contraction in the peripheral nervous system (Chaya et al., 2011).

### SPINAL CORD INJURY (SCI)

The spinal cord connects the CNS to the PNS to control sensory and motor functions. Following spinal cord injury (SCI), this connection can be disrupted to elicit neurological deficits due to impaired transmission between the CNS and PNS. The symptoms of SCI vary widely dependent upon the location and severity of injury but neurological defects can range from pain to paralysis. Given the extensive involvement of Rho GTPases in regulating axonal outgrowth and pathfinding, it is not surprising that members of the Rho GTPase family have emerged as key modulators of axonal regeneration following SCI.

Axonal regeneration following SCI is impeded by increased expression of the inhibitory myelin-associated proteins, Nogo, myelin-associated glycoprotein (MAG), and oligodendrocytes-myelin glycoprotein (OMgp). Early studies in CGNs demonstrated that NogoA and MAG mediate their inhibitory effects on neurite outgrowth through disruption of the delicate balance between antagonistic Rho/ROCK and Rac signaling pathways. In particular, the inhibitory proteins enhance activation of Rho/ROCK signaling and diminish activation of Rac1 (Niederöst et al., 2002). Indeed, in numerous *in vivo* models of SCI, administration of either Rho GTPase inhibitors (e.g., C3 transferase) or ROCK inhibitors (e.g., fasudil, Y-27632) is protective following traumatic nerve injury (Fournier et al., 2003; Boato et al., 2010; Impellizzeri et al., 2010; Boomkamp et al., 2012). *In vivo*, treatment with fasudil improved motor performance while reducing histological damage, the production of proinflammatory cytokines, and activation of caspases following application of vascular clips to the dura via a laminectomy in mice (Impellizzeri et al., 2010). In a similar manner, lentiviral injection of dominant negative ROCK into the adult rat red nucleus protected against damage induced by unilateral rubrospinal tract transection at the fourth cervical level (Wu et al., 2009). Moreover, ROCKII^-/-^ mice subjected to dorsal root nerve crush injury recovered use of the affected forepaw more quickly and demonstrated enhanced regeneration of sensory axons across the dorsal root entry zone when compared to controls (Duffy et al., 2009). Collectively, these studies strongly suggest that enhanced Rho/ROCK signaling impedes axonal growth and regeneration following SCI injury.

In opposition to Rho, recent studies indicate that activation of Rac may be a critical feature which promotes axonal regeneration following SCI. Wallerian degeneration occurs in response to axonal transection and involves axonal degeneration and fragmentation of the myelin sheath. Jung et al. (2011) recently determined that a dominant negative Rac1 mutant or siRNA directed against Rac1 prevents myelin fragmentation *in vivo* following sciatic nerve axotomy in mice. However, constitutively active Rac1 failed to induce myelin fragmentation on uninjured nerves, indicating that Rac1 is an upstream regulator, but is not sufficient to induce myelin degradation following sciatic nerve injury in mice. In contrast to this prior study indicating Rac1 has an essential role in myelin fragmentation following nerve injury, Lorenzetto et al. (2013) demonstrated that injection of cell-penetrating constitutively active Rac1 mutants following optic nerve crush improved survival and axonal regeneration while preventing dendrite degeneration. The pro-survival effects of constitutively active Rac1 may have been mediatated through PAK and ERK1/2 signaling, as both showed enhanced activation in retinal ganglion cells (Lorenzetto et al., 2013). Therefore, although the exact mechanism is presently unclear, these studies indicate that the disrupted balance between Rac and Rho GTPases is a critical determinant of axonal regeneration following traumatic nerve injury.

### ISCHEMIA/REPERFUSION

Cerebral ischemia and reperfusion (I/R) have devastating effects on the brain and increasing evidence suggests that neuronal cell death may be induced by dysregulation of Rho family GTPase activity. In a similar manner to other neurodegenerative diseases and neuronal traumas, recent data indicate that Rac GTPase activity may be dysregulated following I/R. For example, active Rac1 was decreased while total Rac1 redistributed to retracting neuronal processes in the hippocampus 24 h after rats were exposed to global cerebral ischemia (Johanna et al., 2010). This study suggests that aberrant activity and localization of Rac1 may contribute to the axonal retraction and neuronal death following I/R. In other models of I/R, Rac1 has been shown to be elevated in the hippocampus and may delay neuronal cell death through of JNK activation (Zhang et al., 2006, 2009). Alternatively, Rac may also contribute to the release of toxic ROS from microglia in I/R as administration of NSC23766 to rats 15 min prior to induced cerebral ischemia prevented an upregulation in ROS and improved hippocampal-dependent memory and cognitive tests 7–9 days after onset when compared to vehicle-treated rats (Raz et al., 2010). Therefore, although the consequences of enhanced Rac1 activation are presently unclear, dysregulated Rac1 activity occurs in several models of I/R.

In opposition to Rac, Rho is upregulated in the brains of human stroke patients when compared to controls (Brabeck et al., 2003). Further, recent *in vivo* data in rodents has underscored the Rho/ROCK pathway as a significant determinant of neuronal cell death following I/R. Following focal ischemia in mice, expression of Rho is rapidly upregulated in the ischemic hemisphere, peaking between 12 and 24 h after onset and may contribute to neurodegeneration by inducing profound actin cytoskeletal restructuring (Trapp et al., 2001; Erdo et al., 2004). Interestingly, the upregulation of Rho precedes cytoskeletal rearrangements and DNA damage in neurons (Brabeck et al., 2003). Rho appears to exert its pro-death function in neurons through the downstream effector ROCK, as mice exposed to middle cerebral artery occlusion showed a threefold increased expression of ROCK in the ischemic penumbra (Shin et al., 2008), which localized primarily to actin filaments (Yamashita et al., 2007).

Recent pre-clinical studies have examined the therapeutic use of ROCK inhibitors for the treatment of I/R. Administration of the ROCK inhibitor fasudil significantly protected against delayed neuronal death induced by ischemic injury in gerbils, even when administered as late as 24 h after the ischemic insult (Satoh et al., 2007). In a similar manner, fasudil or Y-27632 reduced cerebral infarct size and improved neurologic outcome in mice after middle cerebral artery occlusion (Rikitake et al., 2005). Administration of fasudil following transient cerebral ischemia in rats abrogated an increase in phosphorylated (active) JNK and caspase-3 (Wei et al., 2014). Collectively, extensive data indicate that aberrant activation of the Rho/ROCK pathway underlies neuronal death in I/R.

## CONCLUDING REMARKS

The essential functions of Rho family GTPases in regulating neuronal growth cone dynamics, neurite outgrowth, and neuronal development suggest that Rho GTPases have an important and conserved function in mediating neuronal survival and death. Indeed, although neurodegenerative diseases are complex and multifaceted, dysregulated activity of Rho family GTPases has emerged as a common feature underlying the etiology of diverse degenerative disorders of the central and peripheral nervous systems. Future research will be aimed at further elucidating the signaling pathways that are aberrantly activated or disrupted downstream of Rho GTPases in neurodegenerative diseases. Many studies suggest that targeting Rho GTPases, or their downstream effectors, may have beneficial therapeutic effects for the treatment of neurodegenerative diseases.

## Conflict of Interest Statement

The authors declare that the research was conducted in the absence of any commercial or financial relationships that could be construed as a potential conflict of interest.
